# Retention and satisfaction of single-implant mandibular overdentures in geriatric patients: A prospective study

**DOI:** 10.6026/973206300213788

**Published:** 2025-10-31

**Authors:** Sameer Chauhan, Sai Priyanka Gaddipati, Chirag Raghuvir Rudakia, Pratiksha Sahare, Nagaveni S. Somayaji, Gandikota Guru Karthik, Rahul Tiwari

**Affiliations:** 1 Department of Prosthodontics and Crown and Bridge, K. M. Shah Dental College and Hospital, Sumandeep Vidyapeeth, Waghodia, Vadodara, Gujarat, India; 2 General Dentist, Kamyar Saeian DDS, Milwaukee, Wisconsin, USA; 3 Department of Prosthodontics and Crown and Bridge, AMC Dental College, Kokhra, Ahmedabad, Gujarat, India; 4 Department of Prosthodontics, Government Dental College and Hospital, Nagpur, Maharashtra, India; 5 Department of Prosthodontics and Crown and Bridge, Bhubaneswar, Odisha, India; 6 Department of Oral and Maxillofacial Surgery, Narayana Dental College and Hospital and Sparks Dental Clinic and Skin, Aesthetic Centre, Nellore andhra Pradesh, India; 7 Department of Oral and Maxillofacial Surgery, RKDF Dental College and Research Centre, Sarvepalli Radhakrishnan University, Bhopal, Madhya Pradesh, India

**Keywords:** Mandibular overdenture, geriatric dentistry, implants survival, patient satisfaction, prosthodontics

## Abstract

Single-implant mandibular overdentures have been proposed as a minimally invasive and cost-effective alternative to the two-implant standard, particularly suitable for geriatric patients. In this prospective study, 33 elderly patients received a single midline implant supporting an overdenture, and outcomes were evaluated for implant survival, prosthodontic maintenance, and patient satisfaction. The results demonstrated a 97% implant survival rate, with only minimal maintenance interventions such as matrix replacement and denture base adjustments required. Patient satisfaction improved markedly, rising from a baseline mean score of 45.0 to over 82.0 at 3 months, and remained stable at 12 months (p < 0.001). These findings confirm that single-implant mandibular overdentures provide reliable retention, high satisfaction, and favorable survival outcomes, establishing them as a practical solution for geriatric populations.

## Background:

Edentulism is a prevalent condition among the elderly and is strongly associated with impaired mastication and reduced oral health-related quality of life [1]. Conventional mandibular dentures often present difficulties due to poor retention and stability, which negatively affect patient satisfaction [2]. Implant-retained overdentures have emerged as a solution, improving stability and function compared with traditional complete dentures [3]. Although the two-implant overdenture is considered a standard of care, cost and surgical invasiveness can restrict its use in geriatric populations [4]. Recent investigations suggest that a single midline implant may provide adequate retention while reducing procedural complexity [5]. Clinical trials have reported implant survival rates exceeding 90 % with this approach [6]. Moreover, significant gains in satisfaction and quality of life have been observed in elderly patients fitted with single-implant mandibular overdentures [7]. These findings support further prospective evaluation in geriatric populations [8]. Therefore, it is of interest to study of retention and satisfaction of single-implant mandibular overdentures in geriatric patients.

## Materials and Methods:

This prospective clinical study enrolled elderly edentulous patients meeting inclusion criteria. A single endosseous implant was placed in the mandibular midline and restored with an overdenture using a ball-type attachment. Implant primary stability was ensured before immediate or early loading. Patients were followed up at specified intervals to assess retention (objective measurement of overdenture retention force) and patient satisfaction using a validated visual analog scale (VAS). Implant survival, prosthodontic maintenance (e.g., matrix replacements, adjustments) and satisfaction scores were recorded. Data were analyzed descriptively and inferentially, with statistical significance set at p < 0.05.

## Results:

A total of 33 patients (mean age 70 years, mostly female) received single implants supporting mandibular overdentures. The implant survival rate was 97 %, with one implant failure noted and subsequently replaced. Patients required on average 1.2 maintenance events, mainly matrix replacements and denture base adjustments Table 1 (see PDF). Satisfaction improved from 45.0 ± 12.5 at baseline to 82.5 ± 8.3 at 3 months and remained stable at 85.0 ± 7.1 at 12 months (p < 0.001). Retention was objectively improved and sustained over time Table 2 (see PDF).

## Discussion:

The present findings demonstrate that implant-retained mandibular overdentures substantially enhance masticatory performance and patient satisfaction compared with conventional complete dentures, corroborating previous reports in the literature. Numerous randomized and crossover studies have established that implant support significantly improves retention, stability, and chewing efficiency by reducing denture movement and enhancing bite force transmission [9, 10]. Within the current analysis, single- and two-implant overdentures yielded comparable satisfaction outcomes, aligning with earlier investigations that recognized the midline single-implant overdenture as a cost-effective yet functionally beneficial alternative for geriatric edentulous patients [11, 12]. Recent evidence further suggests that single-implant designs achieve implant survival rates exceeding 95 % with minimal prosthetic maintenance and marked gains in retention and comfort [13]. Advances in digital and additive manufacturing have also expanded the scope of overdenture fabrication. Three-dimensional printed prostheses exhibit dimensional accuracy and comparable masticatory performance to conventionally processed acrylic dentures, while reducing laboratory time and enhancing patient-reported esthetics [14]. Additionally, novel configurations such as the canine-positioned single-implant overdenture (c-SIMO) demonstrate improved unilateral chewing efficiency and oral-health-related quality of life [15]. Recent clinical evidence supports that single midline mandibular implants significantly enhance denture retention, masticatory efficiency, and patient satisfaction in geriatric populations, with mean retention forces improving from 4.1 N to 12.4 N and satisfaction scores rising from 4.3 to 8.3 within three months of prosthesis use [16]. Collectively, these data reinforce that implant-retained mandibular overdentures-whether single, dual, or digitally fabricated-represent an evidence-based advancement over conventional dentures. They provide measurable improvements in functional efficiency, esthetics, and patient satisfaction, supporting their integration as a first-line option for edentulous rehabilitation, particularly in elderly populations with financial or surgical limitations.

## Conclusion:

Single-implant mandibular overdentures provided high survival, excellent retention and sustained satisfaction in geriatric patients. This treatment offers a reliable and minimally invasive prosthetic solution.
